# An audit on smoking status identification and cessation support in a psychiatric inpatient setting

**DOI:** 10.1192/j.eurpsy.2026.10844

**Published:** 2026-07-31

**Authors:** G. Montebello, G. Grech, D. Bonello, M. Galea, K. Callus, C. Purkiss

**Affiliations:** 1 Mount Carmel Hospital, Attard; 2 Mater Dei Hospital; 3 University of Malta, Imsida, Malta

## Abstract

**Introduction:**

Tobacco smoking remains one of the leading causes of preventable death globally, and yet, among psychiatric inpatients, smoking is frequently overlooked or even facilitated.

**Objectives:**

**To assess the documentation** of patients’ smoking history during their first clinical encounter. **To evaluate whether smoking cessation advice/referrals** to cessation services was provided during up until discharge. **To determine if changes in smoking status** were accompanied by documentation of adjustments to psychotropic medication.

**Methods:**

Data collected from files and summary letters from adult patients discharged in December 2024.Data collectors examined if smoking status was documented, if smoking cessation advice was offered, and if referrals to cessation services were recorded. Data was tabulated to determine the proportion of patients with documented smoking status, the identified smokers who received cessation interventions, and the interventions provided—whether pharmacological, behavioral, or via referral. Descriptive statistical methods were run to highlight trends in smoking patterns in patients.

**Results:**

The audit reviewed 219 psychiatric inpatient admissions at Mount Carmel Hospital in December 2024. Smoking status was documented in 87.7% of cases, with 113 patients (51.6%) identified as current smokers. Among these, 85% male, and smoking was significantly associated with male gender (Chi-square p = 0.00041). Smoking was also strongly linked to substance use disorders (F10), with 89% of patients in this group identified as smokers and an odds ratio of 15 (p = 4.89 × 10^-14^). A significant association was observed between smoking and secondary education (p = 0.021), while younger age (20s–30s) was also significantly correlated with smoking (p = 0.00024). Patients with multiple psychiatric diagnoses had nearly twice the odds of smoking compared to those with a single diagnosis, though this was not statistically significant (p = 0.083). No significant associations were found with employment status or nationality.

Critically, smoking cessation support was almost entirely absent. Only one discharge letter mentioned cessation advice or referral, and no clinical documentation indicated any medication adjustments in response to smoking status.

**Image 1:**
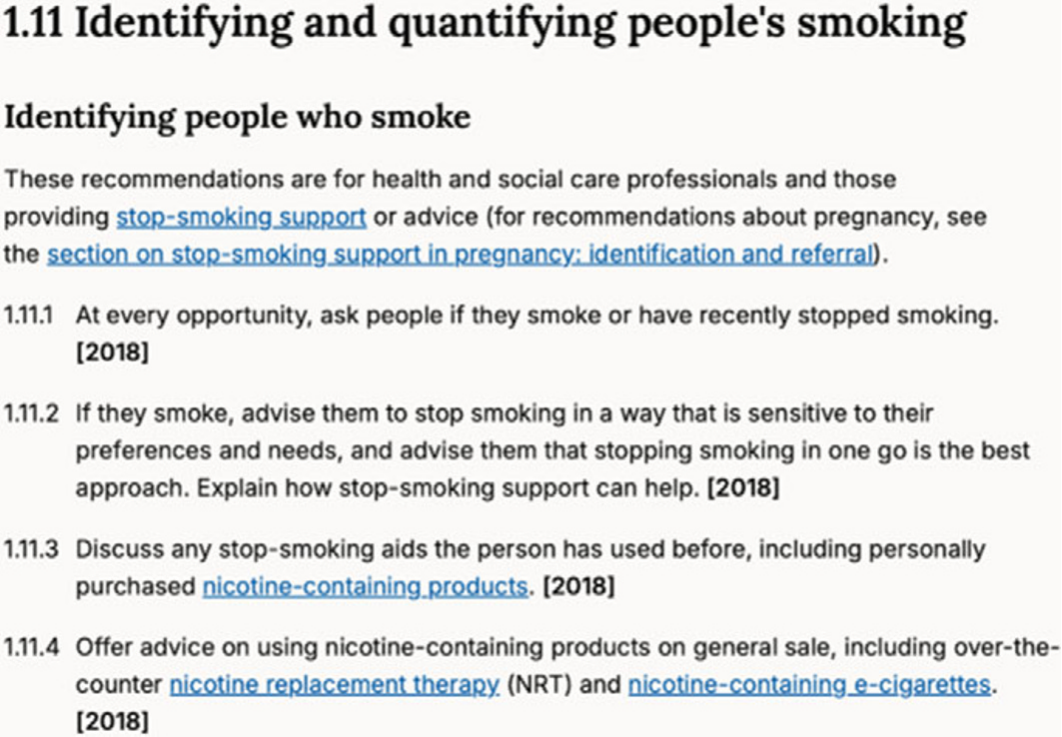
Image 1: Long description.

**Image 2:**
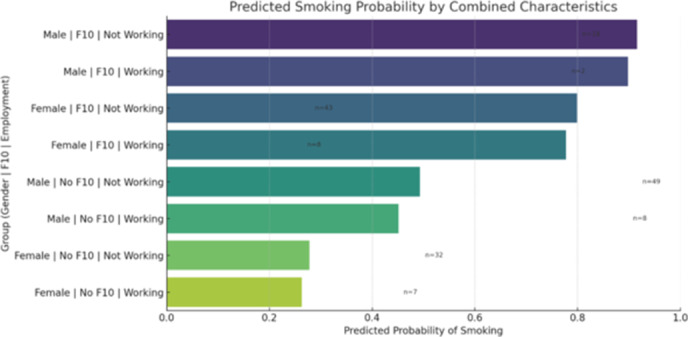
Image 2: Long description.

**Image 3:**
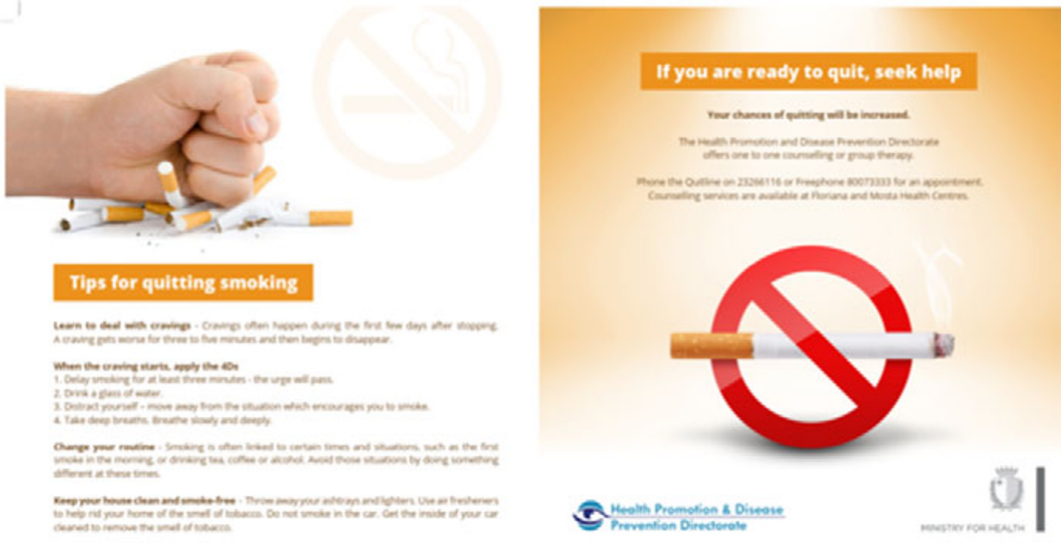
Image 3: Long description.

**Conclusions:**

Nicotine use was documented in 87% of cases but cessation support nearly absent from discharge planning. Significant associations were found between smoking and key demographic and clinical factors, including male gender, younger age, secondary education, substance use disorders (F10), and multiple psychiatric diagnoses. The near-total lack of smoking cessation advice or referrals—alongside the absence of medication reviews in response to smoking status—highlights a critical gap in clinical practice. These findings underscore the need for policies to integrate tobacco use assessment, documentation, and cessation support into routine psychiatric care.

**Disclosure of Interest:**

None Declared

## Long descriptions

### Image 1: Long description

The document contains the following text:

1.11 Identifying and quantifying people's smoking

Identifying people who smoke

These recommendations are for health and social care professionals and those providing stop-smoking support or advice (for recommendations about pregnancy, see the section on stop-smoking support in pregnancy: identification and referral).

1.11.1 At every opportunity, ask people if they smoke or have recently stopped smoking. [2018]

1.11.2 If they smoke, advise them to stop smoking in a way that is sensitive to their preferences and needs, and advise them that stopping smoking in one go is the best approach. Explain how stop-smoking support can help. [2018]

1.11.3 Discuss any stop-smoking aids the person has used before, including personally purchased nicotine-containing products. [2018]

1.11.4 Offer advice on using nicotine-containing products on general sale, including over-the-counter nicotine replacement therapy (N R T) and nicotine-containing e-cigarettes. [2018]

Navigate back to Image 1:.

### Image 2: Long description

The chart title is Predicted Smoking Probability by Combined Characteristics. The y-axis is labeled Group (Gender | F 10 | Employment) and the x-axis is Predicted Probability of Smoking with a scale from 0.0 to 1.0. The bars are color-coded in a gradient from dark purple at the top to lime green at the bottom.

* Male | F 10 | Not Working: Highest probability at approximately 0.92, n equals 18.

* Male | F 10 | Working: Probability at approximately 0.90, n equals 2.

* Female | F 10 | Not Working: Probability at approximately 0.80, n equals 43.

* Female | F 10 | Working: Probability at approximately 0.78, n equals 8.

* Male | No F 10 | Not Working: Probability at approximately 0.50, n equals 49.

* Male | No F 10 | Working: Probability at approximately 0.45, n equals 8.

* Female | No F 10 | Not Working: Probability at approximately 0.28, n equals 32.

* Female | No F 10 | Working: Lowest probability at approximately 0.26, n equals 7.

Navigate back to Image 2:.

### Image 3: Long description

The left panel features a photo of a fist crushing several cigarettes on a white surface. Below this is an orange header titled Tips for quitting smoking. The text includes four main sections. One. Learn to deal with cravings. Two. When the craving starts, apply the 4 Ds: delay smoking for at least three minutes, drink a glass of water, distract yourself, and take deep breaths. Three. Change your routine. Four. Keep your house clean and smoke-free. The right panel has an orange gradient background with a large red no-smoking symbol over a lit cigarette. Above the symbol, text reads: If you are ready to quit, seek help. It states that the Health Promotion and Disease Prevention Directorate offers counseling and group therapy. Contact details include Quitline on 2 3 2 6 6 1 1 6 or Freephone 8 0 0 7 3 3 3 3 for appointments at Floriana and Mosta Health Centres. The bottom right corner features the Ministry for Health crest.

Navigate back to Image 3:.

